# Supplementary material for: Predicting visual function by interpreting a neuronal wiring diagram
Source: Nature. 2024 Oct 2;634(8032):113–23. doi: 10.1038/s41586-024-07953-5 (PMC11446822; doi:10.1038/s41586-024-07953-5)
Supplement: Supplementary file 6 — CRF and ERF predictions for individual TmY4 and TmY9 cells. Analogous to Supplementary Data 3, but for TmY target types. Shown are the top four monosynaptic pathways, the strongest pathway passing through each of the top ten intermediary types (ranking from Extended Data Fig. 7), and the trisynaptic pathway Tm1–TmY–Dm3–TmY (see the section entitled Prediction of spatial normalization). [file 41586_2024_7953_MOESM6_ESM.zip › DataS4/TmY9q/720575940637771583.pdf]

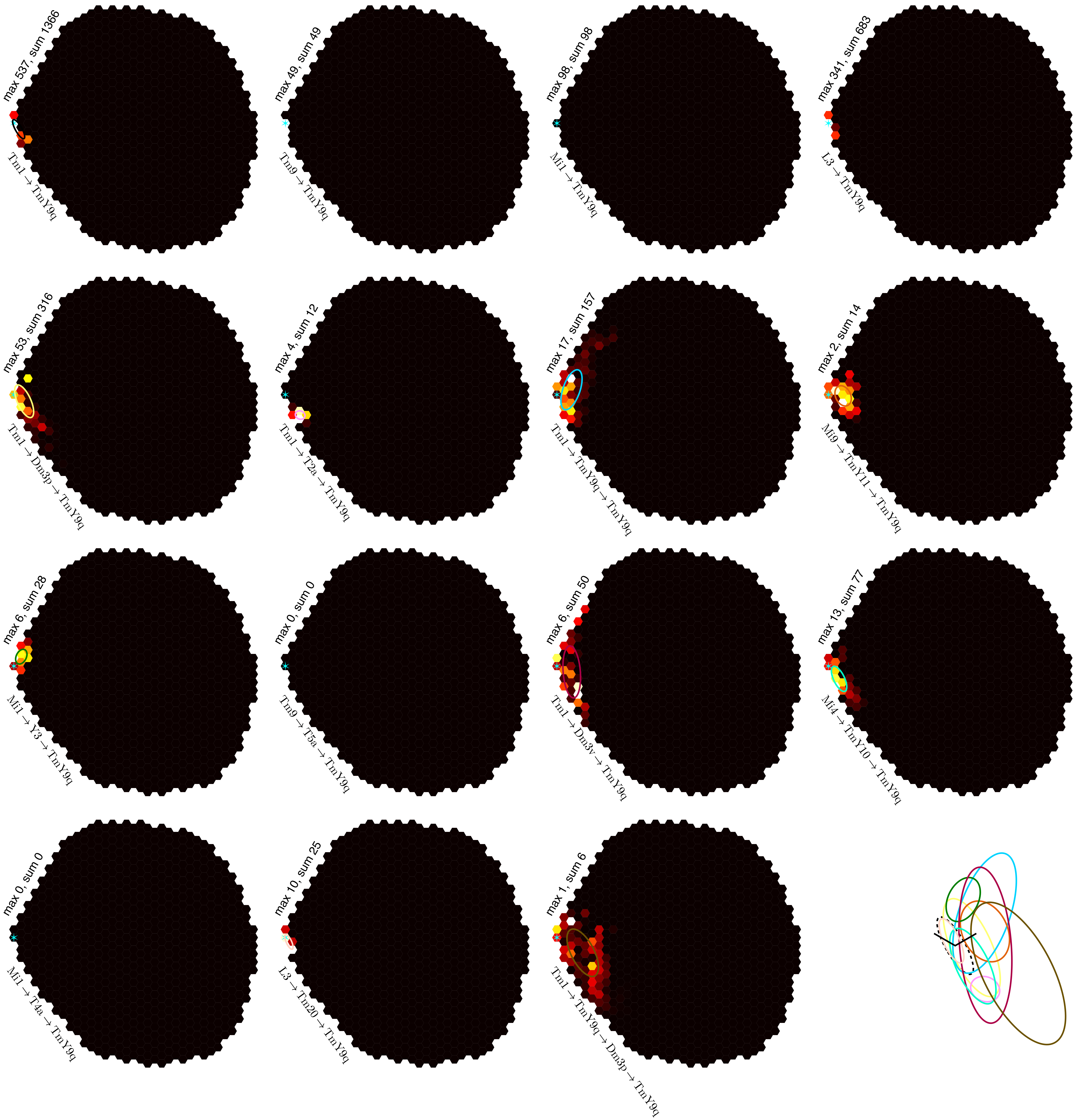

max 537, sum 1366
Tm1 → TmY9q
max 49, sum 49
Tm9 → TmY9q
max 98, sum 98
Mi1 → TmY9q
max 341, sum 683
L3 → TmY9q
max 53, sum 316
Tm1 → Dm3p → TmY9q
max 4, sum 12
Tm1 → T2a → TmY9q
max 17, sum 157
Tm1 → TmY9q → TmY9q
max 2, sum 14
Mi9 → TmY11 → TmY9q
max 6, sum 28
Mi1 → Y3 → TmY9q
max 0, sum 0
Tm9 → T5a → TmY9q
max 6, sum 50
Tm1 → Dm3v → TmY9q
max 13, sum 77
Mi4 → TmY10 → TmY9q
max 0, sum 0
Mi1 → T4a → TmY9q
max 10, sum 25
L3 → Tm20 → TmY9q
max 1, sum 6
Tm1 → TmY9q → Dm3p → TmY9q
